# Efficacy of a Fatty Acids Dietary Supplement in a Polyethylene Glycol-Induced Mouse Model of Retinal Degeneration

**DOI:** 10.3390/nu9101079

**Published:** 2017-09-29

**Authors:** Maurizio Cammalleri, Massimo Dal Monte, Filippo Locri, Emma Lardner, Anders Kvanta, Dario Rusciano, Helder André, Paola Bagnoli

**Affiliations:** 1 Department of Biology, University of Pisa, via San Zeno 31, 56127 Pisa, Italy; maurizio.cammalleri@unipi.it (M.C.); filippo.locri1@gmail.com (F.L.); 2 Interdepartmental Research Center Nutrafood “Nutraceuticals and Food for Health”, University of Pisa, via del Borghetto 80, 56124 Pisa, Italy; 3 Section of Eye and Vision, Department of Clinical Neurosciences, St Erik Hospital, Karolinska Institutet, Polhemsgatan 50, SE-112 82 Stockholm, Sweden; emma.lardner@sll.se (E.L.); anders.kvanta@ki.se (A.K.); helder.andre@ki.se (H.A.); 4 Sooft Fidia Pharma, Contrada Molino 17, 63833 Montegiorgio (FM), Italy; dario.rusciano@sooft.it

**Keywords:** complement system, inflammation, macrophage infiltration, dry age-related macular degeneration, dietary supplementation

## Abstract

Current knowledge of the benefits of nutrition supplements for eye pathologies is based largely on the use of appropriate animal models, together with defined dietary supplementation. Here, C57BL6 mice were subretinally injected with polyethylene glycol (PEG)-400, an established model of retinal degeneration with a dry age-related macular degeneration (AMD)-like phenotype, an eye pathology that lacks treatment. In response to PEG-400, markers of the complement system, angiogenesis, inflammation, gliosis, and macrophage infiltration were upregulated in both retinas and retinal pigment epithelium (RPE)/choroids, whereas dietary supplementation with a mixture based on fatty acids counteracted their upregulation. Major effects include a reduction of inflammation, in both retinas and RPE/choroids, and an inhibition of macrophage infiltration in the choroid, yet not in the retina, suggesting a targeted action through the choroidal vasculature. Histological analysis revealed a thinning of the outer nuclear layer (ONL), together with dysregulation of the epithelium layer in response to PEG-400. In addition, immunohistofluorescence demonstrated Müller cell gliosis and macrophage infiltration into subretinal tissues supporting the molecular findings. Reduced ONL thickness, gliosis, and macrophage infiltration were counteracted by the diet supplement. The present data suggest that fatty acids may represent a useful form of diet supplementation to prevent or limit the progression of dry AMD.

## 1. Introduction

Age-related macular degeneration (AMD) is the leading cause of blindness in the world, and its global prevalence is projected at 196 million by the year 2020 increasing to 288 million in 2040 [1]. Dry AMD accounts for the majority (up to 90%) of AMD cases [2] and is characterized by drusen deposits between the retinal pigment epithelium (RPE) and the choroid. Advanced dry AMD leads to regions of massive RPE death or geographic atrophy (GA). Drusen deposits constitute a chronic inflammatory stimulus further activating drusen biogenesis with consequent lesions of Bruch’s membrane. As a result, some patients advance to wet AMD, with impairment of the RPE layer and invasion of the subretinal space by newly-formed choroidal neovascularization (CNV) [3]. In both cases, advanced AMD culminates in the loss of photoreceptor cells, with subsequent severe visual impairment and, ultimately, blindness.

CNV formation requires continuous and complex interactions among inflammatory factors, cytokines, and the extracellular matrix [4]. In particular, vascular endothelial growth factor (VEGF) is an angiogenic cytokine that plays a decisive role in CNV formation [5], and substantial progress in the development of new therapies for wet AMD has been focused in VEGF inhibition [6]. In contrast, dry AMD remains untreatable [2], although a number of therapeutic options have been developed and are in various stages of clinical trials. Enhancers of choroidal blood flow, neuroprotectors, and anti-complement factors are the most common fronts in the research for pharmacological prevention of dry AMD. In addition, novel therapy options include anti-oxidative or anti-inflammatory compounds, as activation of oxidative stress and inflammatory cascades participate in the pathogenesis of dry AMD. Finally, therapies in the management of dry AMD include nutritional supplements, such as anti-oxidant vitamins or fatty acids that are, at present, extensively investigated in terms of prophylactic benefits, potential harms, and optimal use [7,8]. On the other hand, evidence for the benefit of diet supplementation is often controversial [9]. For instance, epidemiological studies have indicated potential preventive effects of omega-3 fatty acids against dry AMD [10], although the results of the Age-Related Disease Study 2 (AREDS2) seem to exclude their potential in preventing AMD progression [8]. In contrast, issues related to randomized, controlled trials suggest that disregarding the effects of omega-3, and more generally of fatty acids, on the prevention of progression to advanced AMD, may be premature [11].

Designing therapeutic strategies for dry AMD requires the availability of a clinically-relevant animal model in which to understand the mechanisms responsible for the pathology during its initial phases. In this respect, the murine model with subretinal injection of polyethylene glycol (PEG)-400 developed by Lygozubov et al. [12,13] is of unusual interest as it reproduces key pathological events of human AMD. In fact, PEG-400 at 1.0 mg can induce choroidal neovascularization typical of wet AMD [12], while at 0.5 mg mimics drusen formation and leads to degenerative events in the retina and RPE/choroid that characterize dry AMD [13]. In particular, the formation of drusen-like deposits between the RPE and choroid activates the complement system which, in turn, starts a cascade of inflammatory reactions that ultimately triggers degenerative events in the retina and in the RPE/choroid interphase [14], resulting in a reduced thickness of the outer nuclear layer (ONL) accompanied by degenerative events at the photoreceptor and RPE levels. The PEG-400-induced AMD model has been well characterized in terms of inflammation and macrophage response [12]. In addition, C3 and C9 induction with eventual binding to receptors on the macrophage has also been shown [15]. Whether the pathology that characterizes the model can be prevented by therapeutic interventions remains to be explored.

In the present study, the PEG-400 model was used to investigate whether an orally-administered compound based on fatty acids is able to limit the acute inflammatory reaction and macrophage infiltration triggered by the drusen-like insult. The compound is a mixture of fatty acids, lycopene, and spirulina, involved in cell protection from oxidative stress and inflammation [16,17,18]. The effects of this diet supplement were investigated by evaluating markers of the complement system, angiogenesis, inflammation, glia activation, and macrophage infiltration in the retina and in the RPE/choroid of PEG-400-induced mice. Molecular and biochemical data were further correlated with the main landmarks of histological structure and cellular markers.

## 2. Materials and Methods

### 2.1. Animals

This study was carried out in strict accordance with the recommendations in the Guide for the Care and Use of Laboratory Animals of the National Institutes of Health and adheres to the ARVO Statement for the Use of Animals in Ophthalmic and Vision Research. Procedures were carried in compliance with the Italian guidelines for animal care (DL 116/92) and the European Communities Council Directive (86/609/EEC), and are approved by the Commission for Animal Wellbeing of the University of Pisa (Permit Number: 0009069/2014). All efforts were made to reduce both animal suffering and the number of animals used.

Two-month-old C57BL/6J male mice, used in accordance with Lyzogubov et al. [12,13], were originally purchased from Charles River Laboratories Italy (Calco, Italy) and mated in-house to a breeding colony. Seventy-eight mice were used for this study. Mice were housed in a regulated environment (23 ± 1 °C, 50 ± 5% humidity) with a 12 h light/dark cycle (lights on at 08.00 a.m.), and provided with a standard diet and water ad libitum.

### 2.2. Subretinal Injection of PEG

Subretinal injection of PEG-400 was performed in agreement with Lyzogubov et al. [13]. Sixty-one mice were injected with 0.5 mg of PEG in 2 μL PBS. Subretinal bleb formation was considered as successful subretinal injection. Mice were euthanized six days after PEG-400 injection and eyes were collected. To reduce the number of mice used in the study, we did not perform subretinal injections with vehicle, as PBS did not induce damage to retinal cells or the retinal structure [13].

### 2.3. Dietary Supplementation

The diet supplement used in this study is commercialized in tablets by Sooft Fidia Pharma (Montegiorgio, Italy) as Macular-FAG (from now on referred as mFAG). Each tablet (1650 mg of weight) contains 550 mg of a patented mixture of saturated and unsaturated fatty acids (including palmitic, oleic, stearic, linoleic, and azelaic acids), referred as fatty acid group (FAG; Again Life Italia s.r.l., Schio, Italy), supplemented with 200 mg of spirulina (from *Spirulina platensis*) and 1 mg of lycopene (from *Solanum lycopersicum* L.). There is evidence that, in cultured human monocytes, FAG is non-toxic up to 1 mg/mL and inhibits lipopolysaccharide-induced release of inflammatory cytokines [19]. One-hundred mg of mFAG was suspended in 2 mL of 10% sucrose in water (vehicle). Two-hundred μL of the suspension, containing 10 mg of mFAG, was daily administered to PEG-400-injected mice by oral gavage. This dose corresponds to the recommended in humans (1–2 tablets/70 kg daily), normalized by the body surface area method for interspecies’ drug dosage translation [20]. mFAG was administered according to three different regimens: for 10 days before PEG-400 injection (pre-PEG-400); for five days after PEG-400 injection (post-PEG-400); and for 10 days before and five days after PEG-400 injection (pre- and post-PEG-400). Mice non-induced with PEG-400, nor mFAG, were used as normal control (from here on referred to as naive), while vehicle was orally administered to PEG-400-injected mice according to the pre- and post-PEG-400 paradigm. Fourteen mice were used in the vehicle group, while 6, 6, and 23 mice were used in the pre-PEG-400, post-PEG-400, and pre- and post-PEG-400 groups, respectively. A schematic diagram depicting the experimental groups is shown in Figure S1.

### 2.4. Quantitative Real Time PCR

Mice were deeply anesthetized and euthanized by cervical dislocation. Retinas and RPE/choroid complexes were rapidly dissected and stored at −80 °C until use for molecular analyses.

Quantitative real-time PCR (qPCR) experiments were performed using six independent samples, each containing two retinas or two RPE/choroids from two mice, per experimental condition. Total RNA was extracted using RNeasy Mini Kit (Qiagen, Valencia, CA, USA). First-strand cDNA was generated from 1 μg of total RNA (QuantiTect Reverse Transcription Kit, Qiagen, Valencia, CA, USA). Real-time PCR amplification was performed with SsoAdvanced Universal SYBR Green Supermix (Bio-Rad Laboratories, Hercules, CA, USA) on a CFX Connect Real-Time PCR detection system and software CFX manager (Bio-Rad Laboratories, Hercules, CA, USA). qPCR primer sets for the complement proteins C3 and C5, VEGF, tumor necrosis factor-α (TNF-α), interleukin (IL)-1β, IL-6, IL-8, intercellular adhesion molecule-1 (ICAM-1), the inducible form of nitric oxide synthase (iNOS), glial fibrillary acidic protein (GFAP), cluster of differentiation 68 (CD68), and endothelial growth factor-like module-containing mucin-like hormone receptor-like 1 (F4/80) were chosen to hybridize to unique regions of the appropriate gene sequence (see Table S1 for a complete list of assayed genes and primers). Amplification efficiency was near 100% for each primer pair (Opticon Monitor 3 software, Bio-Rad Laboratories, Hercules, CA, USA). Target genes were assayed concurrently with Rpl13a, a gene encoding for ribosomal protein L13A. Samples were compared using the relative threshold cycle (Ct Method). The increase or decrease (fold change) was determined relative to naive mice after normalization to Rpl13a. All reactions were performed in triplicate.

### 2.5. Enzyme-Linked Immunosorbent Assays

Quantification of C3, C5, VEGF, TNF-α, IL-1β, IL-6, IL-8, CD68, and F4/80 protein levels was performed using commercially available kits (LifeSpan Biosciences, Inc., Seattle, WA, USA for C3, C5, CD68, and F4/80; R&D Systems, Minneapolis, MN, USA for VEGF, TNF-α, IL-1β, and IL-6; MyBioSource, San Diego, CA, USA for IL-8). Protein levels were evaluated in six independent samples, as described in qPCR. Samples were lysed with RIPA lysis buffer (50 mM Tris, pH 7.4 containing 150 mM NaCl, 1% Triton X-100, 1% sodium deoxycholate, 0.1% sodium-dodecyl sulphate (SDS), 5 mM ethylene-diaminetetracetic acid (EDTA) and proteinase inhibitor cocktail (Roche Applied Science, Indianapolis, IN, USA). Protein content was quantified by the Micro BCA Protein Assay (Thermo Fisher Scientific, Waltham, MA, USA). Enzyme-linked immunosorbent assay (ELISA) plates were evaluated spectrophotometrically (Microplate Reader 680 XR, Bio-Rad Laboratories, Hercules, CA, USA) according to the manufacturers’ instructions. Data were expressed as picograms of targets per milligram of protein. All experiments were performed in duplicate.

### 2.6. Western Blot

Western blot analysis was performed using the protein extracts from ELISA quantifications. Aliquots of each sample containing equal amounts of protein (30 μg) were subjected to SDS-polyacrylamide gel electrophoresis, and β-actin was used as loading control. Gels were transblotted onto a polyvinylidene difluoride membrane, and the blots were blocked in 3% skim-milk for 1 h at room temperature, followed by incubation overnight at 4 °C with a primary goat polyclonal antibody directed to ICAM-1 (1:200 dilution; sc-1511; Santa Cruz Biotechnology, Santa Cruz, CA, USA), or with primary rabbit polyclonal antibody directed to iNOS (1:200 dilution; sc-8310; Santa Cruz Biotechnology, Santa Cruz, CA, USA) or GFAP (1:200 dilution; sc-9065; Santa Cruz Biotechnology, Santa Cruz, CA, USA). Finally, blots were incubated for one hour at room temperature with HRP-conjugated secondary antibodies (1:5000; Santa Cruz Biotechnology, Santa Cruz, CA, USA) and developed with Clarity Western enhanced chemiluminescence substrate (Bio-Rad Laboratories, Inc., Hercules, CA, USA). Images were acquired (ChemiDoc XRS^+^; Bio-Rad Laboratories, Inc., Hercules, CA, USA), and the optical density (OD) of the bands was evaluated (Image Lab 3.0 software, Bio-Rad Laboratories, Inc., Hercules, CA, USA). The data were normalized to the corresponding OD of β-actin. All experiments were performed in duplicate.

### 2.7. Tissue Processing for Histology

Mice were deeply anesthetized and euthanized by perfusion-fixation with 4% paraformaldehyde (PFA) in PBS, eyeballs were enucleated, post-fixed for 24 h, and subsequently processed for paraffin embedding. Microtome sections were obtained from 24 eyeballs from 24 different mice (five naive, eight PEG-400-injected, and 11 PEG-400-injected receiving a diet supplemented with mFAG in the pre- and post-PEG-400 paradigm). Sections were deparaffinized, and 4 μm sections were subjected to morphometric analysis, while 6 μm sections were used for immunohistochemistry.

For morphometric analysis, images of hematoxylin and eosin (H and E) stained sections were acquired near the site of lesion between the optic nerve and the ciliary body, using an Axioskop 40 microscope (Zeiss, Gottingen, Germany) coupled to a VisiCam TC10 (VWR, Lutterworth, UK). The site of lesion was identified as a small scleral damage caused by needle penetration. Measurements of ONL thickness were acquired per slide, as previously described [13], and corrected versus total retinal thickness, using ImageJ (Version 1.47, NIH freeware, Bethesda, MD, USA). All experiments were performed in duplicate.

For immunohistochemistry, deparaffinized eye section were processed for antigen retrieval with EDTA buffer, pH 9, for 20 min at 100 °C, and immune-reactions were performed in a Bond III robotic system (Leica Biosystems, Newcastle, UK). Primary antibodies: mouse monoclonal anti-RPE65; mouse monoclonal anti-S100A8/A9 complex (MAC387); and goat monoclonal anti-GFAP (all Abcam, Cambridge, UK; cat. No. ab78036, ab22506, and ab207165, respectively). Secondary antibodies: donkey anti-mouse Alexa Fluor 647 conjugated; and goat anti-rabbit Alexa Fluor 546 conjugated (both Thermo Fisher Scientific, Waltham, MA, USA; cat. No. A31571 and A11010, respectively). Eye sections were counterstained with Hoechst 33258 (Sigma Aldrich Corp., St. Louis, MO, USA; cat. no. 14530). Images were acquired on an Axioskop 2 plus fluorescence microscope with the AxioVision software (Version 4.6, Zeiss, Gottingen, Germany).

### 2.8. Statistical Analysis

All data were analyzed by the Shapiro-Wilk test to certify normal distribution. Statistical significance was evaluated with GraphPad Prism 5 software (GraphPad Software, Inc., San Diego, CA, USA) using unpaired Student’s *t*-test or one-way ANOVA followed by the Newman-Keuls multiple comparison post-hoc test, as appropriate. After statistical analysis, the data from different experiments were plotted and averaged in the same graph. Results were expressed as the mean ± S.E.M. of the indicated *n* values. Differences with *p* < 0.05 were considered significant.

## 3. Results

### 3.1. Characterization of the Experimental Model

**Figure 1:**
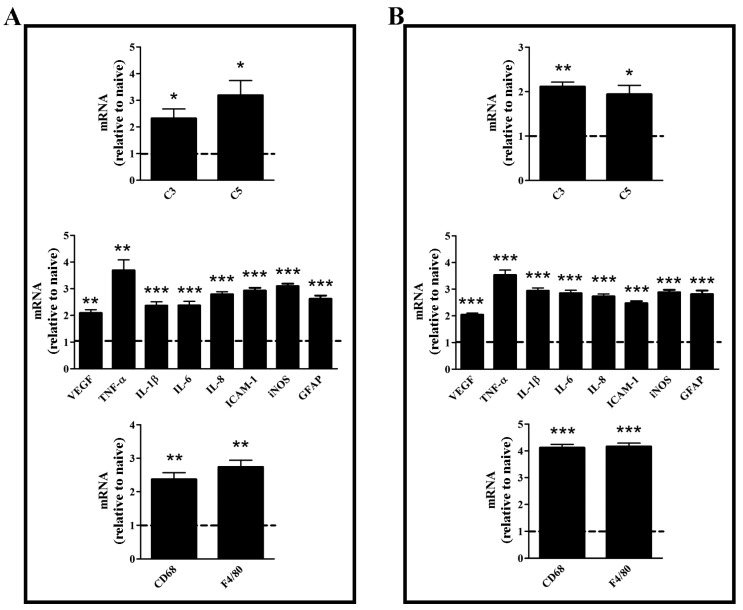
Transcript levels of markers of the complement system, angiogenesis, inflammation, and macrophage infiltration in the retina (**A**) or in the retinal pigment epithelium (RPE)/choroid (**B**). Transcripts were evaluated by relative quantification with quantitative real time PCR (qPCR) in mice untreated or subretinally injected with 0.5 mg polyethylene glycol (PEG-400). Data were analyzed by the formula 2^−ΔΔCT^ using ribosomal protein L13A (Rpl13a) as the internal standard, and presented as the mean ± S.E.M. (*n* = 6/group). * *p* < 0.05, ** *p* < 0.01, and *** *p* < 0.001 versus naive (unpaired Student’s *t*-test).Dotted lines, mRNA levels in naive.

PEG-400-induced mice displayed significantly increased transcript levels of markers of the complement system, angiogenesis, inflammation, and macrophage infiltration both in the retina (Figure 1A) and the RPE/choroid (Figure 1B). Comparable results were found in PEG-400 mice whose diet was supplemented with vehicle (data not shown). C3 and C5 are markers of the alternative pathway of the complement system that, once activated, causes an overproduction of angiogenic and inflammatory cytokines, including VEGF, TNF-α, and IL-1β, IL-6, and IL-8. In particular, PEG-400-induced mice displayed an increase at the transcript level of C3 and C5 of about 2.3- and 3.2-fold (*p* < 0.05) in the retina, and 2.1- (*p* < 0.01), and 2.9-fold (*p* < 0.05) in the RPE/choroid. The angiogenic and inflammatory transcripts VEGF, TNF-α, IL-1β, IL-6, and IL-8 were consequently increased in the retina by about 2.1- (*p* < 0.01), 3.7- ( *p* < 0.01), 2.4- (*p* < 0.001), 2.4- (*p* < 0.001), and 2.8-fold (*p* < 0.001), while an increase of 2.0-, 3.5-, 2.9-, 2.8-, and 2.7-fold (*p* < 0.001) was observed in the RPE/choroid. In addition, an increased expression of ICAM-1 and iNOS by about 2.9- and 3.1-fold (*p* < 0.001) in the retina, and 2.5- and 2.9-fold (*p* < 0.001) in the RPE/choroid was denoted. Moreover, a significant increase was observed for GFAP, a protein localized to the end-feet and processes of astrocytes and Müller cells, and a biomarker of gliosis. GFAP transcript levels increased in PEG-400-induced mice by about 2.6-fold (*p* < 0.001) in the retina, and 2.5-fold (*p* < 0.001) in the RPE/choroid. Finally, PEG-400 caused macrophage infiltration as demonstrated by the increased levels of CD68 and F4/80, both membrane proteins expressed by macrophages, of which F4/80 is specifically expressed by murine macrophages [21]. In particular, CD68 and F4/80 increased by about 2.4- and 2.7-fold (*p* < 0.01) in the retina, and 4.1- and 4.2-fold (*p* < 0.001) in the RPE/choroid.

### 3.2. Effects of Dietary Supplementation with mFAG: Molecular and Biochemical Analysis

**Figure 2:**
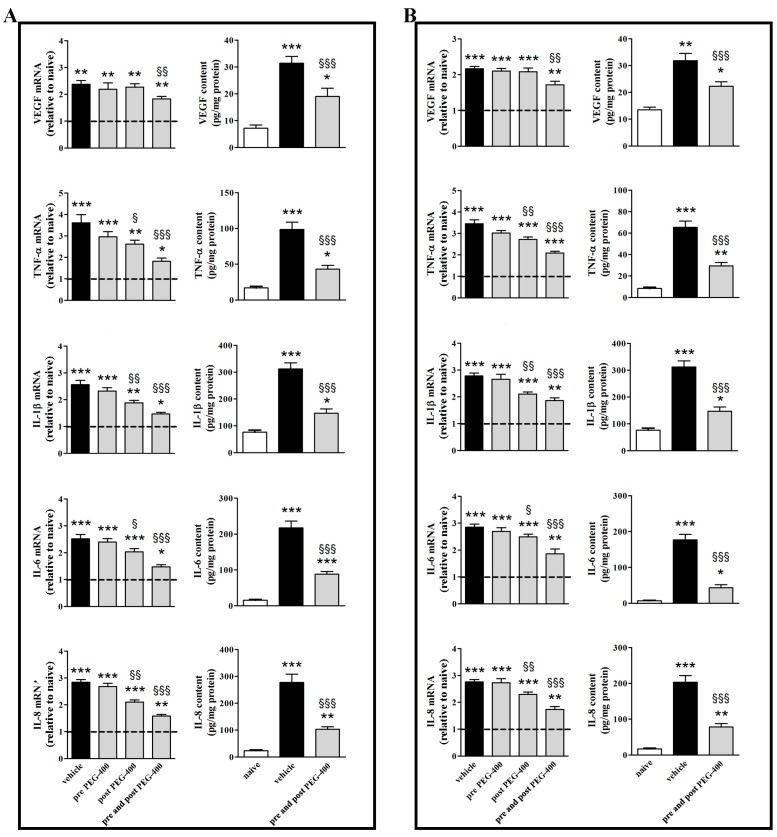
Macular-FAG (mFAG) dietary supplementation reduces PEG-400-induced upregulation of markers of angiogenesis and inflammation in the retina (**A**) or in the RPE/choroid (**B**). Levels were evaluated in mice untreated or subretinally injected with 0.5 mg PEG-400 and receiving vehicle or mFAG for 10 days before PEG-400 injection (pre-PEG-400), for five days after PEG-400 injection (post-PEG-400), or for 10 days before and five days after PEG-400 injection (pre- and post-PEG-400). Transcripts were evaluated by relative quantification with qPCR. Data were analyzed by the formula 2^−ΔΔCT^ using Rpl13a as the internal standard. Proteins were evaluated by ELISA (LifeSpan Biosciences, Inc., Seattle, WA, USA). Data were presented as the mean ± S.E.M. (*n* = 6/group). * *p* < 0.05, ** *p* < 0.01, and *** *p* < 0.001 versus naive. ^§^ *p* < 0.05, ^§§^ *p* < 0.01, and ^§§§^ *p* < 0.001 versus vehicle (one-way ANOVA followed by the Newman-Keuls multiple comparison post-hoc test). Dotted lines, mRNA levels in naive; white bars, naive; black bars, PEG-400-induced mice with vehicle dietary supplementation; grey bars, PEG-400-induced mice with mFAG dietary supplementation.

**Figure 3:**
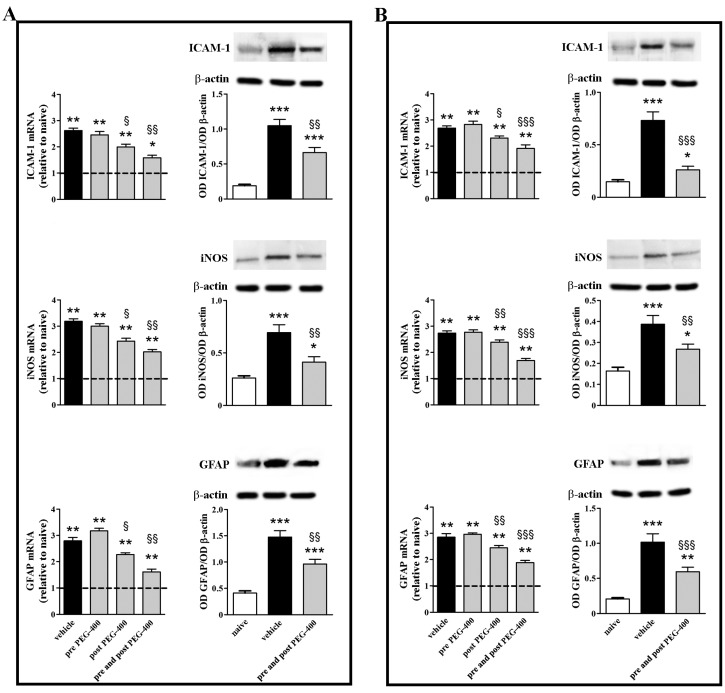
mFAG dietary supplementation reduces PEG-400-induced upregulation of markers of inflammation in the retina (**A**) or in the RPE/choroid (**B**). Levels were evaluated in mice untreated or subretinally injected with 0.5 mg PEG-400 and receiving vehicle or mFAG for 10 days before PEG-400 injection (pre-PEG-400), for five days after PEG-400 injection (post-PEG-400), or for 10 days before and five days after PEG-400 injection (pre- and post-PEG-400). Transcripts were evaluated by relative quantification with qPCR. Data were analyzed by the formula 2^−ΔΔCT^ using Rpl13a as the internal standard. Proteins were evaluated by Western blot using β-actin as loading control. Data were presented as the mean ± S.E.M. (*n* = 6/group). * *p* < 0.05, ** *p* < 0.01 and *** *p* < 0.001 versus naive; ^§^ *p* < 0.05, ^§§^ *p* < 0.01 and ^§§§^ *p* < 0.001 versus vehicle (one-way ANOVA followed by the Newman-Keuls multiple comparison post-hoc test). Dotted lines, mRNA levels in naive; white bars, naive; black bars, PEG-400-induced mice with vehicle dietary supplementation; grey bars, PEG-400-induced mice with mFAG dietary supplementation.

**Figure 4:**
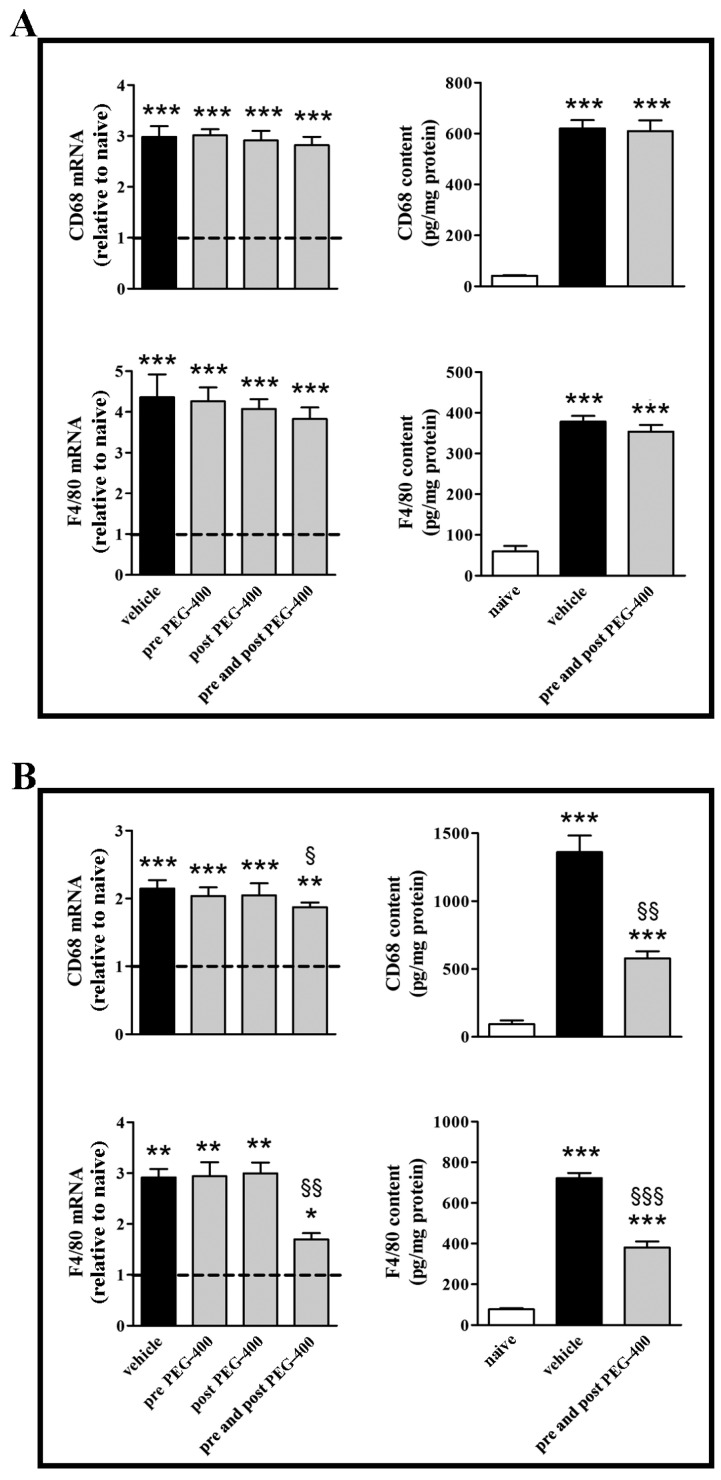
mFAG dietary supplementation reduces the PEG-400-induced upregulation of markers of macrophage infiltration in the retina (**A**) or in the RPE/choroid (**B**). Levels were evaluated in mice untreated or subretinally injected with 0.5 mg PEG-400 and receiving vehicle or mFAG for 10 days before PEG-400 injection (pre-PEG-400), for five days after PEG-400 injection (post-PEG-400), or for 10 days before and five days after PEG-400 injection (pre- and post-PEG-400). Transcripts were evaluated by relative quantification with qPCR. Data were analyzed by the formula 2^−ΔΔCT^ using Rpl13a as the internal standard. Proteins were evaluated by ELISA. Data were presented as the mean ± S.E.M. (*n* = 6/group). * *p* < 0.05, ** *p* < 0.01, and *** *p* < 0.001 versus naive; ^§^ *p* < 0.05, ^§§^ *p* < 0.01, and ^§§§^ *p* < 0.001 versus vehicle (one-way ANOVA followed by the Newman-Keuls multiple comparison post-hoc test). Dotted lines, mRNA levels in naive; white bars, naive; black bars, PEG-400-induced mice with vehicle dietary supplementation; grey bars, PEG-400-induced mice with mFAG dietary supplementation.

Figure 2, Figure 3 and Figure 4 show the effects of diet supplementation with mFAG on upregulated biomolecular markers in the retina (Figure 2A, Figure 3A, and Figure 4A) and the RPE/choroid (Figure 2B, Figure 3B, and Figure 4B), as evaluated at the transcript and protein levels. In both the retina and the RPE/choroid, PEG-400-induced upregulation of C3 and C5 was not influenced by mFAG irrespectively of the supplementation regimen (data not shown). As shown in Figure 2, mFAG, if supplemented pre- or post-PEG-400, did not affect VEGF transcripts in either the retina or the RPE/choroid. VEGF transcript and protein reduction required pre-PEG-400 mFAG supplementation and continuing post-PEG-400, in both the retina and the RPE/choroid. Post-PEG-400 mFAG supplementation significantly reduced transcript levels of TNF-α, IL-1β, IL-6, and IL-8, both in the retina and RPE/choroid, but the inhibitory effect of the diet supplement was stronger if mFAG was administered both pre- and post-PEG-400. The efficacy of this regimen was confirmed by the drastic reduction of protein levels of TNF-α, IL-1β, IL-6, and IL-8. In particular, pre- and post-PEG-400 mFAG supplementation reduced VEGF transcripts by approximately 1.3-fold (*p* < 0.01) in both retinas and RPE/choroids. At the protein level, after PEG-400 VEGF increased by about 4.4-fold (*p* < 0.001) in the retina and 1.7-fold (*p* < 0.01) in the RPE/choroid, pre- and post-PEG-400 mFAG supplementation reduced VEGF to approximately 1.7-fold (*p* < 0.05) in both the retina and the RPE/choroid. Post-PEG-400 mFAG supplementation reduced TNF-α, IL-1β, IL-6, and IL-8 transcripts by nearly 1.4- (*p* < 0.05), 1.4- (*p* < 0.01), 1.2- (*p* < 0.05), and 1.4-fold (*p* < 0.01) in the retina, and 1.3- (*p* < 0.01), 1.3- (*p* < 0.01), 1.1- (*p* < 0.05), and 1.2-fold (*p* < 0.01) in the RPE/choroid. Pre- and post-PEG-400 mFAG supplementation reduced TNF-α, IL-1β, IL-6, and IL-8 transcripts by roughly 2.0-, 1.7-, 1.7-, and 1.8-fold (*p* < 0.001) in the retina, and 1.7-, 1.5-, 1.5-, and 1.6-fold (*p* < 0.001) in the RPE/choroid. At the protein level, after PEG-400 TNF-α, IL-1β, IL-6, and IL-8 increased to approximately 5.9-, 4.1-, 14.2-, and 12.0-fold (*p* < 0.001) in the retina, and 7.8-, 3.0-, 24.6-, and 11.9-fold (*p* < 0.001) in the RPE/choroid. Pre- and post-PEG-400 mFAG supplementation reduced TNF-α, IL-1β, IL-6, and IL-8 by about 2.3-, 2.1-, 2.5-, and 2.7-fold (*p* < 0.001) in the retina, and 2.2-, 1.7-, 4.1-, and 2.6-fold (*p* < 0.001) in the RPE/choroid.

As illustrated in Figure 3, post-PEG-400 mFAG supplementation significantly reduced transcript levels of ICAM-1, iNOS, and GFAP, in both the retina and the RPE/choroid. The inhibitory effect of mFAG was more evident if mFAG was administered both pre- and post-PEG-400. The efficacy of this regimen was confirmed by the drastic reduction of protein levels of ICAM-1, iNOS, and GFAP, as observed for transcript levels. In particular, post-PEG-400 mFAG supplementation reduced ICAM-1, iNOS, and GFAP transcripts by nearly 1.3-, 1.3- and 1.2-fold (*p* < 0.05) in the retina, and 1.2- (*p* < 0.05), 1.1- (*p* < 0.01), and 1.2-fold (*p* < 0.01) in the RPE/choroid. Moreover, pre- and post-PEG-400 mFAG supplementation reduced ICAM-1, iNOS, and GFAP transcripts by about 1.7-, 1.6-, and 1.7-fold (*p* < 0.01) in the retina, and 1.4-, 1.6-, and 1.5-fold (*p* < 0.001) in the RPE/choroid. At the protein level, after PEG-400 ICAM-1, iNOS, and GFAP increased by approximately 5.5-, 2.7-, and 3.6-fold (*p* < 0.001) in the retina, and 5.0-, 2.4- and 4.9-fold (*p* < 0.001) in the RPE/choroid. Pre- and post-PEG-400 mFAG supplementation reduced ICAM-1, iNOS, and GFAP to about 1.6-, 1.7- and 1.5-fold (*p* < 0.01) in the retina, and 2.8- (*p* < 0.001), 1.4- (*p* < 0.01) and 1.7-fold (*p* < 0.001) in the RPE/choroid.

In the retina (Figure 4A), mFAG supplementation did not affect transcript or protein levels of the macrophage markers CD68 and F4/80, irrespectively of the supplementation regimen. In contrast, in the RPE/choroid (Figure 4B), mFAG was able to significantly reduce the levels of CD68 and F4/80 transcripts and proteins, yet only if supplemented with both pre- and post-PEG-400. Specifically, in the RPE/choroid, pre- and post-PEG-400 mFAG supplementation reduced CD68 and F4/80 transcripts by about 1.1- (*p* < 0.05) and 1.7-fold (*p* < 0.01). At the protein level, after PEG-400 CD68 and F4/80 increased to nearly 14.7- and 9.3-fold (*p* < 0.001) in the RPE/choroid, and pre- and post-PEG-400 mFAG supplementation reduced CD68 and F4/80 to approximately 2.4- (*p* < 0.01) and 1.9-fold (*p* < 0.001).

### 3.3. Effects of Dietary Supplementation with mFAG: Structural and Immunohistochemical Analysis

**Figure 5:**
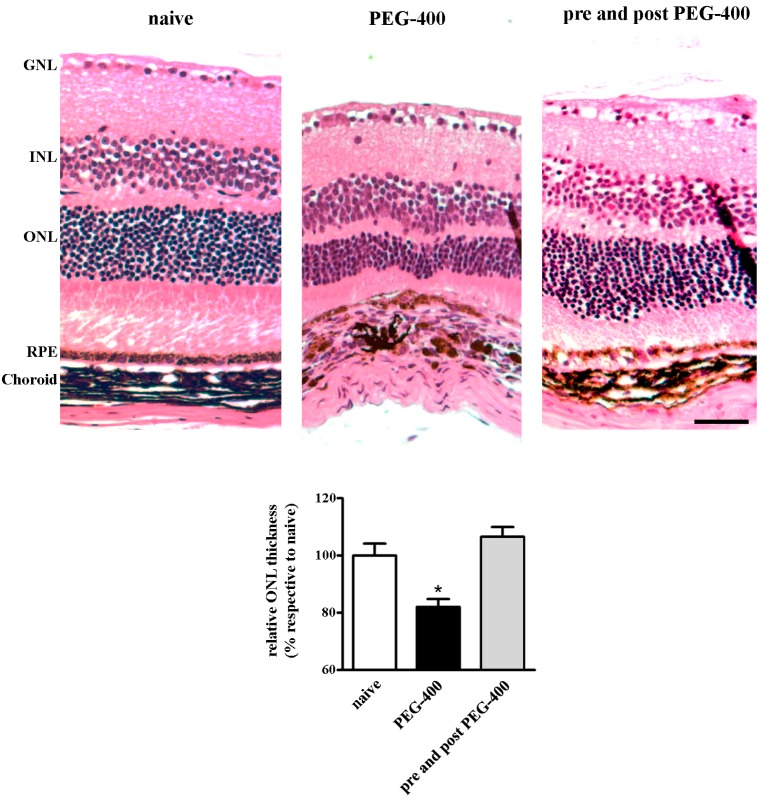
mFAG dietary supplementation ameliorates PEG-400-induced retinal damage. Illustrative H and E images of eyes from mice untreated (naive) or subretinally injected with 0.5 mg PEG-400 in the absence (PEG-400) or presence (pre- and post-PEG-400) of mFAG dietary supplementation pre- and post-PEG-400. Measurements of the ONL thickness were normalized to total retinal thickness and plotted versus naive, as the mean ± S.E.M. (*n* = 5, 8, or 11, respectively per group). * *p* < 0.05 (one-way ANOVA followed by Newman-Keuls multiple comparison post-hoc test). Scale bar = 50 μm. Retinal layers: GNL, ganglion nuclear layer; INL, inner nuclear layer; ONL, outer nuclear layer; RPE, retinal pigment epithelium.

In general terms, when compared to either pre- or post-PEG-400, pre- and post-PEG-400 mFAG dietary supplementation produced stronger reduction of molecular levels of angiogenesis and inflammatory cytokines induced by PEG-400. Based on these observations, retinal morphological analysis was focused on PEG-400 retinal damage, in the absence or presence of pre- and post-PEG-400 mFAG dietary supplementation. Representative images of H and E stained retinas and RPE/choroids are shown in Figure 5 in which morphometric analysis of the outer retina displayed a significant thinning of the ONL (approaching 20%, *p* < 0.05), accompanied with a considerable dystrophic RPE layer upon PEG-400 damage. mFAG dietary supplementation resulted in a normal thickness of the ONL (statistically undistinguishable from naive) and a noticeable recovery of the RPE cells.

**Figure 6:**
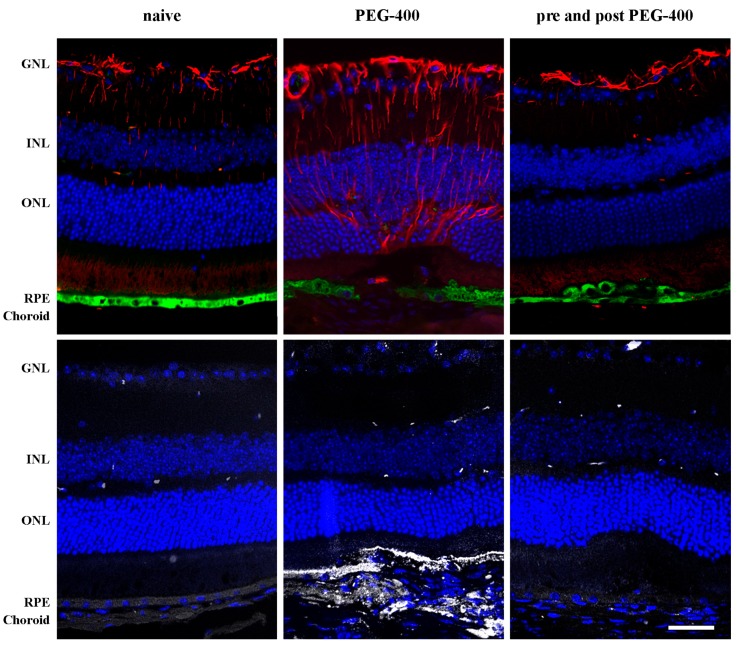
Dietary supplementation with mFAG normalizes overexpression of PEG-400-induced retinal damage tissue markers. Representative immunohistochemistry images of mice eyes as described in Figure 5. Panels represent glial fibrillary acidi protein- GFAP-)positive Müller cells (**red**), RPE65 specific staining of RPE cells (**green**), membrane attack complex (MAC) staining for activated macrophages (**white**), and the nuclei were counterstained with Hoechst 33342 (**blue**). Scale bar = 50 μm. Retinal layers: GNL, ganglion nuclear layer; INL, inner nuclear layer; ONL, outer nuclear layer; RPE, retinal pigment epithelium.

PEG-400-induced retinal damage, as a hallmark of the dry AMD-like mouse model, has been previously described as an atrophy to the RPE [13], and could be confirmed by a dramatic decrease of RPE65 immunostaining (Figure 6), upon PEG-400 damage. Moreover, increased GFAP staining to the processes of PEG-400-induced retinal Müller cells confirmed the previously described mRNA and protein findings (Figure 1 and Figure 3). Macrophage recruitment was analyzed by staining activated macrophages (Figure 6), and a denoted increase in the macrophage marker was present in PEG-400-induced mice, particularly in the choroid. It is noteworthy that RPE atrophy, Müller cells gliosis, and macrophage recruitment were normalized in PEG-400-induced mice with mFAG dietary supplementation (Figure 6).

## 4. Discussion

Nutritional supplements are widely taken by the general population and several of such products are marketed specifically to improve ocular health, despite controversies in their use in clinical ophthalmology [22]. The results of the present study demonstrate that a diet supplement based on fatty acids was, indeed, effective in counteracting the dry AMD-like pathological signs that characterize the PEG-400-induced retinal degeneration mouse model.

Against a background of considerable epidemiological and other evidence implicating omega-3 fatty acids in the prevention of AMD progression, the negative results of the AREDS2 study were debated in light of the possibility that the design, setting, intake, or subjects of the study might not have permitted the prophylactic potential of omega-3 to be adequately demonstrated [11]. On the other hand, there is evidence that in addition to omega-3 and omega-6 fatty acids also influence gene expression, therefore, acting as a candidate to interact with the complex molecular cascades involved in AMD [23]. A final consideration is that the balance of omega-6/omega-3 fatty acids is important for health. For instance, fish intake has been reported to lower the risk of AMD, especially when omega-6 fatty acid intake was reduced [24]. In a study involving twins, Seddon et al. [25] showed that fish consumption in combination with omega-3 fatty acid intake reduces the risk of AMD. In the present case, mFAG consists mostly in a mixture of fatty acids including omega-3 and omega-6. The present findings suggest that mFAG contains a healthy ratio of these essential fatty acids although its exact composition is covered by a patent. In this respect, there is evidence that a diet containing fatty acids in a low omega-6/omega-3 ratio (2:1 or 3:1) reduces inflammatory processes, while a diet with a high ratio, such as the Western diet (averagely 16:1), promotes the pathogenesis of many diseases, including inflammatory diseases [26]. In addition, mFAG contains lycopene and spirulina that may synergize with fatty acids in reducing retinal and RPE/choroid degeneration in the PEG-400 model. In fact, it has been reported that lycopene reduces the production of inflammatory markers, including ICAM-1, in human RPE cells [27]. In addition, spirulina is rich in xanthophyll zeaxanthin, which has been associated with a reduced risk of AMD in humans [28]. The food supplement mFAG has been present in the Italian market for about one year, with a main recommendation for patients with early signs of atrophic macular degeneration with visible drusen. Currently, it is estimated that approximately 4000 subjects are using mFAG, and one clinical study is under way in Italy at four independent ophthalmology clinics. The clinical trial comprehends the primary endpoints related to the amount of drusen and the area of atrophic retina measured by OCT, expecting that patients treated with mFAG show decreased disease progression. Secondary endpoints are visual acuity and the visual analogic scale test to measure subjective improvements. Since no treatments with demonstrated efficacy for atrophic AMD are available on the market, mFAG presents a critical, unmet clinical need, with promising expectations in delaying AMD progression in humans.

As shown by the present results, the subretinal PEG-400 mouse model is characterized by upregulated levels of factors involved in the complement system, inflammation, and angiogenesis, suggesting deregulation of strictly interconnected pathways that co-dependently participate in AMD pathogenesis. In particular, drusen deposits, located between the RPE basal lamina and the elastin-containing Bruch’s membrane, contain several complement proteins, including C3 and C5, indicating the possibility for local complement-mediated inflammation [29]. In the PEG-400 dry AMD-like mouse model, complement upregulation of C3 and C5 is most likely dependent on interference with regulatory proteases. Therefore, PEG-400 may potentially impair the control that proteases exert on the complement system [30], thus contributing to the formation of the terminal complement. Hence, activation of the complement receptors on the RPE by PEG-400-upregulated C3 and C5 participates in the pathological lesions observed in the outer retina and Bruch’s membrane [31]. Upregulation of the complement system found here is in line with previous results in models of dry AMD [13,32], and is in agreement with the finding that mice lacking of CD46, a membrane cofactor protein that plays a key role in the complement alternative pathway [33], demonstrate spontaneous age-related degenerative changes in the retina, RPE, and choroid that are consistent with the cardinal features of human AMD [34]. Increased levels of the complement system would cause an accumulation of inflammatory factors, which feed-back and activate the complement system, and lead to a drastic macrophage infiltration. Macrophage infiltration further activates inflammatory processes with an additional activation of the complement system [35,36]. Moreover, complement activation stimulates RPE cells to release VEGF [37]. Additional VEGF production is mediated by ICAM-1, yet VEGF itself acts as an inflammatory molecule by promoting ICAM-1 expression. ICAM-1 overexpression leads to activation of leukocytes and release of inflammatory cytokines, most of which are produced by gliotic Müller cells within the retina [38]. As shown here, PEG-400-induced drusen-like insult promotes Müller cell gliosis, as demonstrated by the marked increase in GFAP expression. Moreover, the significant increase of inflammatory cytokines illustrated here is in agreement with previous results [13]. Among the upregulated inflammatory cytokines, TNF-α plays a predominant role in inflammatory responses and is produced by several cells of the retina and the choroid, including macrophages and retinal neurons [39,40]. TNF-α is responsible also for the production of ILs that play a primary role in neovascular pathologies of the retina and choroid [41,42,43,44]. In particular, IL-1β activates IL-6 production, and can act upstream of iNOS. Consequently, the increase in iNOS here observed could participate in ICAM-1 upregulation and subsequent AMD-associated leukostasis [45]. Overall, upregulated complement system together with marked inflammation, gliosis, and oxidative stress participate in macrophage infiltration, a major event contributing to RPE and photoreceptor loss [4].

As shown by the present results, mFAG exerts a multitarget role by interfering with major events activated by PEG-400, here illustrated by a general reduction of pro-inflammatory and angiogenic cytokines associated with AMD. In addition, the fact that effects of mFAG depend on the dietary supplementation protocol and that mFAG efficacy differs between the retina and RPE/choroid adds further evidence to a specificity of action of mFAG dietary supplementation. In the biomolecular cascade activated by PEG-400, mFAG has been found to reduce inflammation and macrophage infiltration, without interfering with the upregulation of the complement system. This is in line with the concept that the complement system is difficult to modulate with small molecules, although anti-complement therapies are under investigation for use in AMD [46], in agreement with the findings presented here of increased C3 and C5 in the dry AMD-like mouse model.

Both the retina and the choroid are similarly responsive to mFAG in terms of the reduction of inflammation, which suggests a major anti-inflammatory role of the dietary supplementation. On the other hand, the finding that the choroid, but not the retina, is responsive to mFAG with a significant reduction of macrophage infiltration markers CD68 and F4/80 is indicative of major effects of mFAG supplementation on macrophage recruitment at the choroidal level. In this respect, specific macrophage phenotypes are associated with wet AMD and macrophages from AMD patients are more pathogenic as compared with controls [47]. In that manner, mFAG could exert effects relevant for wet AMD, in addition to the effects on dry AMD here described. In addition, the macrophage markers used here did not allow us to distinguish between specific macrophages phenotypes, yet the data presented indicates a possibility that mFAG targets, more specifically, macrophages infiltrating the choroid. Nonetheless, some consideration should be given to the possibility that the distribution of dietary mFAG to the choroid was higher than to the retina, therefore, justifying the different efficacy in the choroid as compared to the retina.

At the structural level, PEG-400-induced retinal damage correlates well with previously published data [13], where a dystrophic RPE layer leads to a significant thinning of the ONL of the retina. Interestingly, pre- and post-PEG-400 dietary supplementation with mFAG results in recovery of the ONL thickness, with clear benefits to the RPE cells. Effects of PEG-400-induced retinal damage are further confirmed by immunofluorescence. As illustrated by RPE65 staining, cellular damage similar to geographic atrophy can be observed in PEG-400-induced retinal damage. Moreover, GFAP staining of PEG-400-damaged retinas display an increase in gliotic Müller cells, as suggested by the molecular analysis with increased GFAP transcript and protein levels. Importantly, mFAG dietary supplementation beneficial effects on RPE cells are confirmed by RPE65 immunostaining, with a partial recovery of the RPE layer, and consequent reduction of gliotic Müller cells, as observed by the reduction of GFAP staining through the retina. Upon PEG-400 induction, an increase in messenger and protein levels of C3, C5, CD68, and F4/80 is observed. A central event of complement activation and macrophage recruitment is the deposition of the membrane attack complex (MAC) on the RPE and choroidal cells [48]. In fact, CD68 and F4/80 are significantly downregulated in mFAG supplemented PEG-400-induced mice RPE/choroidal complexes, while not being reduced in the retina. When immunostained with antibodies for activated macrophages, an increase is observed in PEG-400-induced retinal damage, followed by a recovery in mFAG-supplemented animals. In addition, MAC deposition from activated macrophages and monocytes has been shown to contribute to the synthesis and secretion of VEGF, a major growth factor responsible for CNV [49]. These findings may suggest that mFAG supplementation has choroid-specific effects, putatively relevant for wet AMD.

Mechanisms underlying the protective action of mFAG are difficult to postulate and their elucidation needs additional work. One possibility is that fatty acid mixture supplementation reinstates the deficiency of anti-inflammatory bioactive lipids that play a predominant role in AMD [50]. In this respect, polyunsaturated fatty acid (PUFA) administration has been shown to counteract several neovascular pathologies of the eye through different actions. For instance, omega-3 PUFA supplementation to preterm infants may suppress retinopathy of prematurity (ROP) by reinstating correct levels of substances that are decreased in retinal diseases, as is the example of serum adiponectin in ROP [51]. Additionally, fatty acids may act by favoring the production of neurotrophic factors that are needed for the survival of retinal neurons [52]. On the other hand, lipid deposits in AMD have been shown to accelerate disease pathogenesis and reducing the lipid burden by statins is a desirable therapeutic strategy [53]. However, epidemiological studies have reported conflicting results regarding the efficacy of statins in AMD progression [54]. In addition, aging effects on retinal responses to PEG-400 either alone or in combination with diet supplementation with mFAG needs to be further explored. Age-related changes associated with disease contribute not only to disease’s development, but also to how the eye will respond to therapeutic intervention. In particular, aging increases not only the frequency and severity of deposits under the RPE, but also the capacity of dietary fat to induce sub-RPE deposits, especially in the presence of environmental light [55] or in response to laser photochemical injury [56].

**Figure 7:**
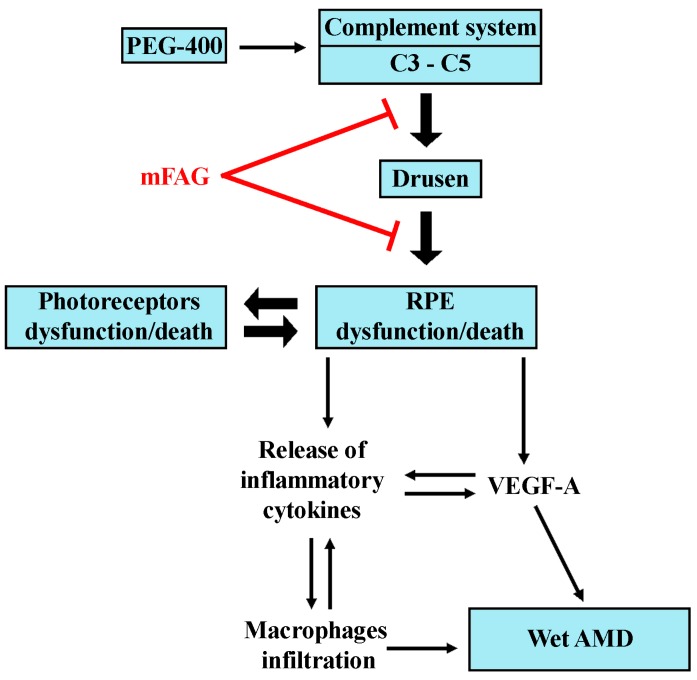
Schematic diagram depicting a possible mechanism of action of mFAG. mFAG, by interfering with complement-mediated activation of RPE cells, would modulate the synthesis and secretion of AMD-associated cytokines and reduce macrophage infiltration in the retina and the choroid, thus preventing or limiting the progression of AMD to the wet form.

Taken together, the present findings suggest that mFAG exerts a predominant anti-inflammatory action both in the retina and in the choroid both in the case when supplementation is given only post- and when given pre- and post-PEG-400 induction of retinal damage. In agreement with the data presented here, that both C3 and C5 are upregulated in the dry AMD-like mouse model, this findings suggests that mFAG may interfere with complement-mediated activation of RPE cells, therefore, modulating synthesis and secretion of AMD-associated cytokines (Figure 7). The additional finding that mFAG specifically targets the choroid in terms of macrophage infiltration is indicative of its effect in preventing choroidal MAC-dependent VEGF stimulation, an established cause of CNV [49].

## 5. Conclusions

The results presented here show the first evidence of dietary supplementation efficacy in the PEG-400 mouse model of retinal damage. Furthermore, the presented data suggest that mFAG dietary supplementation, particularly pre- and post-PEG-400-induced retinal damage, could be beneficial for prevention and/or progression of advanced AMD. We postulate that mFAG causes reduced complement-mediated effects, thus decreasing macrophage recruitment and modulating the production of pro-inflammatory and angiogenic cytokines which, in turn, improves degenerative events in the retina associated with advanced AMD. Of interest is the observation of choroid-specific effects of mFAG dietary supplementation in the dry AMD-like mouse model, which could have implications on the transition to CNV. Although the mechanistic relationship between mFAG and the PEG-400 retinal damage model is preliminary, the present work lays the groundwork for investigations to clarify these issues. Understandably, future studies on how a food supplement may ameliorate some pathological signs of ocular pathologies will require further work and the use of additional experimental models. In further investigations, it would also be of interest to know if the effects of mFAG are limited to the PEG-400 model or may be extended to additional models, such as laser-induced CNV, thus generalizing the effect of mFAG diet supplementation as a preventive form of treatment for AMD.

## Supplementary Materials

The following are available online at www.mdpi.com/2072-6643/9/10/1079/s1. Figure S1: Schematic diagram depicting the experimental groups involved in the study; Table S1: Sequences of primer sets used for qPCR experiments.
