# Supplementary material for: Efficacy of a Fatty Acids Dietary Supplement in a Polyethylene Glycol-Induced Mouse Model of Retinal Degeneration
Source: Nutrients. 2017 Sep 29;9(10):1079. doi: 10.3390/nu9101079 (PMC5691696; doi:10.3390/nu9101079)
Supplement: Supplementary file 1 [file nutrients-09-01079-s001.zip › nutrients-221246-supplementary.pdf]

**Figure S1.** Schematic diagram depicting the experimental groups involved in the study.

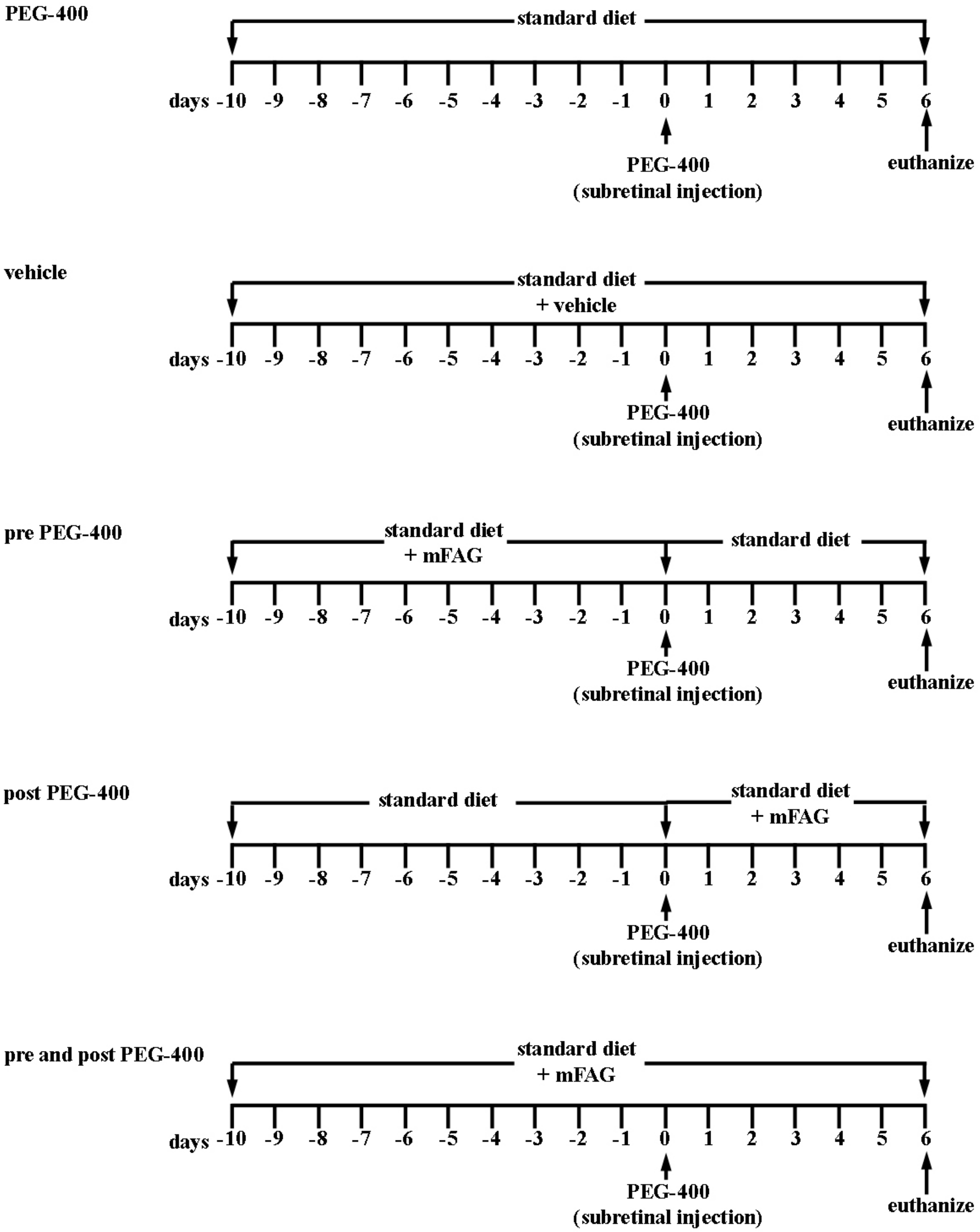

**Table S1.** Sequences of primer sets used for qRT-PCR experiments.

| Gene | Primer sequence (5′ –> 3′) | |
|---|---|---|
| | **Forward Primer** | **Reverse Primer** |
| *C3* | CCAGCTCCCCATTAGCTCTG | CCAGCTCCCCATTAGCTCTG |
| *C5* | GAACAAACCTACGTCATTTCAGC | GTCAACAGTGCCGCGTTTT |
| *VEGF* | GCACATAGGAGAGATGAGCTTCC | CTCCGCTCTGAACAAGGCT |
| *TNF-α* | GCCTCTTCTCATTCCTGCTT | CTCCTCCACTTGGTGGTTTG |
| *IL-1β* | TCCTTGTGCAAGTGTCTGAAGC | ATGAGTGATACTGCCTGCCTGA |
| *IL-6* | TCTGCAAGAGACTTCCATCCAGT | TCTGCAACTGCATCATCGTTGT |
| *IL-8* | ACTTTCAGAGACAGCAGAGC | GTGGTCCACTCTCAATCACT |
| *ICAM-1* | CGCTGTGCTTTGAGAACTGTG | ATACACGGTGATGGTAGCGGA |
| *iNOS* | GGCAAACCCAAGGTCTACGTT | TCGCTCAAGTTCAGCTTGGT |
| *GFAP* | CGGAGACGCATCACCTCTG | AGGGAGTGGAGGAGTCATTCG |
| *CD68* | TGTCTGATCTTGCTAGGACCG | GAGAGTAACGGCCTTTTTGTGA |
| *F4/80* | TGACTCACCTTGTGGTCCTAA | CTTCCCAGAATCCAGTCTTTCC |
| *Rpl13a* | CACTCTGGAGGAGAAACGGAAGG | GCAGGCATGAGGCAAACAGTC |
